# Dr. Marshall Hall

**Published:** 1857-11

**Authors:** 


### NECROLOGY.

Died, on the 11th of August, at Brighton, England, of inanition, consequent upon stricture of the œsophagus, DR. MARSHALL HALL, in the 67th year of his age.

Dr. Hall's fame as a physiologist and philosophic physician is well known to the medical profession throughout all parts of the civilized world, and however much difference of opinion there may be concerning his merits as a man of true genius, all must acknowledge that he was an eminently active, enthusiastic, zealous cultivator of scientific medicine. Of this the number and character of his writings are sufficient proof.

Dr. Hall was born in Nottinghamshire, in the year 1790. His father was a manufacturer of cotton goods, a man of excellent mental endowments, of comfortable means, and of a respectable position in society.

Having received his general education at Nottinghamshire, and some instructions in medicine and chemistry at Newark, Dr. Hall went to Edinburgh, in 1809, and matriculated in the University, then in the noontide of its resplendent reputation. Here he took his degree in 1812, and having been immediately afterward appointed to the post of house-physician at the Royal Infirmary, commenced a course of lectures the ensuing year on the Principles of Diagnosis. He left Edinburgh in 1814, on a visit to the continent, and there travelled, mostly on foot, from one seat of medical learning to another, making himself personally acquainted with the various celebrities of Paris, Berlin, Giessen, Gottingen, and the other less distinguished cities of France and Germany. In 1815 he returned to his native place, and settled down for the practical pursuit of his profession. His success in obtaining business was unusually rapid and profitable, but urged by a higher ambition, he removed to London in 1826. During this time, however, he had made many physiological and pathological investigations, and in 1824 he read before the Royal Medical and Chirurgical Society, a paper, detailing his "Observations on Bloodletting, founded on Researches on the Morbid and Curative Effects of the Loss of Blood."

Having taken up his residence in the great metropolis, he continued his experiments upon the blood, directed now, however, more especially to the circulation of this fluid through the capillaries. In the course of these studies, his attention was accidentally directed to some of the phenomena of nervous action, as exhibited in the bodies and limbs of decapitated animals, and after repeated experiments, he announced the discovery of a new principle in nervous power, which he denominated "the excito-motory or reflex action." This is generally looked upon as his greatest achievement, although it is supposed by many that he was indebted to Prochaska for much that he claimed as his own. Since that time, up to the day of his death, his labors were devoted mainly to the study of the physiology and pathology of the nervous system, although, in the meanwhile, he lent his powers to the elucidation of many other questions connected with medicine and the collateral sciences, and cultivated the practice of his profession so successfully as to realize an ample fortune.

In 1853, Dr. Hall made a visit to the United States and Canada, and we have personal knowledge of the great surprise that he felt at finding the profession of our interior cities so fully acquainted with all the important medical facts recently suggested or established by our European brethren. In this, as in many other matters not immediately belonging to his sphere of study, he exhibited a remarkable degree of unsophistication, as is amply proved by his venturing to advise our people, through the public prints, in regard to one of our politico-social institutions, of which he was evidently as ignorant as the veriest of London cockneys.

Dr. Hall's contributions to the literature of medicine are numerous and varied. Besides his memoirs upon "Bloodletting," "On the Reflex Function of the Medulla Oblongata," "On the True Spinal Marrow and the Excito-Motor System of Nerves," and several papers on kindred subjects, presented to the Royal Society or communicated to the journals, he published several separate works, of which the principal are, "The Principles of Diagnosis," "The Effects of Irritation and Exhaustion after Parturition," "Commentaries on the Diseases of Females," "An Experimental Essay on the Circulation of the Blood," "The Principles of the Theory and Practice of Medicine," "Lectures on the Nervous System," "A Treatise on the Diseases and Derangements of the Nervous System," and "A Treatise on Epilepsy."

